# Transcriptome Analysis of *Otodectes cynotis* in Different Developmental Stages

**DOI:** 10.3389/fmicb.2022.687387

**Published:** 2022-04-04

**Authors:** Ran He, Qian Zhang, Xiaobin Gu, Yue Xie, Jing Xu, Xuerong Peng, Guangyou Yang

**Affiliations:** ^1^Department of Parasitology, College of Veterinary Medicine, Sichuan Agricultural University, Chengdu, China; ^2^Department of Chemistry, College of Life and Basic Science, Sichuan Agricultural University, Chengdu, China

**Keywords:** *Otodectes cynotis*, SMRT, Illumina, different expressed genes, allergen

## Abstract

The mite *Otodectes cynotis* is distributed worldwide and parasitism the ear canals of cats and dogs, causing otitis externa. Molecular biology of *O. cynotis* is poorly understood, with only a few genes being deposited in public databases. In the present study, we aimed to perform transcriptome analysis of *O. cynotis* using SMRT and Illumina sequencing of RNA from different development stages. SMRT-Seq of *O. cynotis* demonstrated 5,431 final transcripts, including 406 long non-coding RNAs and 2,698 differentially expressed genes (DEGs), including 1,357 up-regulated genes and 1,341 down-regulated genes between adult mites and nymph/larva. A total of 397 putative allergen genes were detected, 231 of which were DEGs. Among them, 77 were homologous of known mite allergens. The expression level of allergen genes hints at the pathogenicity of mites in different life stages, and the protein interaction network analysis could identify possible key genes in the pathogenic mechanism. Intriguingly, Gene Ontology analysis showed that most of the (DEGs) were associated with the terms hydrolase activity and proteolysis. Kyoto Encyclopedia of genes and genomes (KEGG) analysis identified drug metabolism-cytochrome P450 signal pathway as one of the top pathways. SMRT-Seq of the full-length transcriptome of *O. cynotis* was performed first, and a valuable resource was acquired through the combination analysis with the Illumina sequencing data. The results of our analyses provide new information for further research into *Otodectes cynotis*.

## Introduction

**FIGURE 1:**
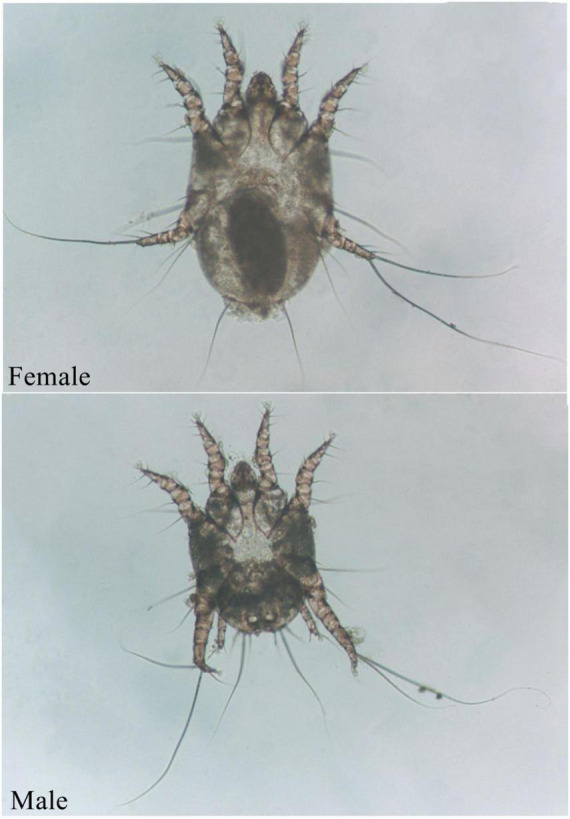
Adult *O. cynotis* mites.

The ear mite *O. cynotis* is the most common etiological agent of otitis externa in cats and dogs (Bosco et al., 2019; Silva et al., 2020). It also parasitizes in the ear canal of ferrets, red foxes, and terrestrial carnivores (Taenzler et al., 2017; Briceño et al., 2020; Fanelli et al., 2020; Huang et al., 2020). Animals can be infected by direct or indirect contact with infected animals (Six et al., 2016). A brown waxy substance can be found in the ear canal of mildly infested cats, which then form a crust on the surface of the ear canal. As the irritation intensifies, itching becomes more and more obvious. Cats keep shaking their heads, scratching their ears, and rubbing their ears on objects due to the itching, resulting to hematomas and ulcers (Montoya, 2018). Cats may cramp or move in circles in severe cases. In addition, purulent otitis externa can be caused by the occurrence of secondary bacterial infections (Roy et al., 2011). The life cycle of *O. cynotis* is incomplete metamorphosis (egg, larva, nymph, and adult) (Tonn, 1961). The mite is long-legged and can be easily recognized as whitish spots in the ear canal. Underside of its body, the chitinous bars behind the either front legs meet to form a V shape, and the female mites have suckers only on the front two pairs of legs (Figure 1). *O. cynotis* mites live on the surface of the ear canal of cats and dogs (Curtis, 2004). They pierce the host skin with its mouthparts, and feed on the lymph, tissue fluid, and blood of the host (Powell et al., 1980), they cause stimulation of the parasitic site, leading to dermatitis or allergic reactions (Montoya, 2018), causing excessive keratinization and proliferation of epithelial cells (Powell et al., 1980). Meanwhile, inflammatory cells, especially mast cells and macrophages increase, and subcutaneous blood vessels dilate (Kelleher, 2001).

Current research on *O. cynotis* focuses mainly on case reports, drug treatments, and *in vitro* acaricidal (Carithers et al., 2016; Yang and Huang, 2016). Only a few studies of its molecular biology (Salib and Baraka, 2011; Taenzler et al., 2017). As a result, there are only a limited number genes from *O. cynotis* mites deposited in public databases (Hu et al., 2019). In the present study, we aim to use single-molecule real-time sequencing (SMRT-Seq) and Illumina based RNA-sequencing (RNA-Seq) to obtain sequence information of *O. cynotis* mites, analyze the expression of allergen genes at different life stages, and determine the protein-protein interaction (PPI) network of *O. cynotis* mites.

## Materials and Methods

### Ethics Approval and Consent to Participate

This study was approved by the Animal Care and Use Committee of Sichuan Agricultural University (SYXK2019-187). All animal procedures used in this study were carried out in strict accordance with the guide for the Care and Use of Laboratory Animals (National Research Council, Bethesda, MD, United States) and the recommendations of the ARRIVE guidelines.^1^ All methods were carried out in accordance with relevant guidelines and regulations.

### Sample Collection

*O. cynotis* mites were isolated from the crust in the ear canal of naturally infested cats. Cats were obtained from the cattery in Chengdu City. The veterinarian treated cats accordingly after the crusts were taken. Crusts were incubated in petri dishes at 35°C, and mites were collected every half an hour. Live mites (larvae, nymphs, and adults) were identified depending on their morphological criteria (Ahn et al., 2013). Samples that included larva, nymph, and adult mites (Ocy_A_N_L) were utilized for SMRT-Seq Meanwhile, nymph/larva mite samples (Ocy_N_L) and adult mite samples (Ocy_A) were prepared for Illumina sequencing. Each group comprised three samples.

Total RNA was isolated using RNeasy Plus Mini Kit (Qiagen, Valencia, CA, United States), the mites were homogenized by full grinding. The purity and concentration of the RNA were evaluated using a NanoDrop ND-1000 spectrophotometer (NanoDrop Technologies, Rockland, DE, United States) using the OD260/280 ratio. The integrity of RNA was determined using agarose gel electrophoresis on an Agilent 2100 Bioanalyzer (Agilent Technologies, Santa Clara, CA, United States).

### Single-Molecule Real-Time Sequencing

The Iso-Seq library of the Ocy_A_N_L group was prepared according to the Isoform Sequencing protocol using the Clontech SMARTer PCR cDNA Synthesis Kit and the BluePippin Size Selection System protocol as described by Pacific Biosciences (PacBio) (PN 100-092-800-03; Pacific Biosciences, Menlo Park, CA, United States). Full length cDNAs were obtained, subjected to DNA damage repair and end repair, and then ligated to sequencing adapters and digested with exonuclease. Libraries that met the quality criteria were sequenced on the PacBio Sequel platform according to the effective concentration and data output requirements of the library.

### Illumina Sequencing

For the Ocy_N_L and Ocy_A groups, a total amount of 1.5 μg RNA per sample was used as input material for the RNA sample preparations. Sequencing libraries were generated using NEBNext^®^ Ultra™ RNA Library Prep Kit for Illumina^®^ (NEB, United States) following manufacturer’s recommendations and index codes were added to attribute sequences to each sample. Briefly, mRNA was purified from total RNA using poly-T oligo-attached magnetic beads. Fragmentation was carried out using divalent cations under elevated temperature in NEBNext First Strand Synthesis Reaction Buffer (5X). First strand cDNA was synthesized using random hexamer primer and M-MuLV Reverse Transcriptase (RNase H^–^). Second strand cDNA synthesis was subsequently performed using DNA Polymerase I and RNase H. Remaining overhangs were converted into blunt ends *via* exonuclease/polymerase activities. After adenylation of 3′ ends of DNA fragments, NEBNext Adaptor with hairpin loop structure were ligated to prepare for hybridization. In order to select cDNA fragments of preferentially 250∼300 bp in length, the library fragments were purified with AMPure XP system (Beckman Coulter, Beverly, United States). Then 3 μl USER Enzyme (NEB, United States) was used with size-selected, adaptor-ligated cDNA at 37°C for 15 min followed by 5 min at 95°C before PCR. Then PCR was performed with Phusion High-Fidelity DNA polymerase, Universal PCR primers and Index (X) Primer. At last, PCR products were purified (AMPure XP system) and library quality was assessed on the Agilent Bioanalyzer 2100 system. The clustering of the index-coded samples was performed on a cBot Cluster Generation System using TruSeq PE Cluster Kit v3-cBot-HS (Illumina) according to the manufacturer’s instructions. After cluster generation, the library preparations were sequenced on an Illumina Hiseq platform NovaSeq 6000 (Illumina, San Diego, CA, United States) and 125 bp/150 bp paired-end reads were generated. The fastp (0.19.7, -g -q 5 -u 50 -n 15 -l 150) was used to conduct quality control on Illumina raw data. Raw reads of fastq format were firstly processed through in-house perl scripts. In this step, clean reads were obtained by removing reads containing adapter, reads containing ploy-N and low-quality reads from raw reads. At the same time, Q20, Q30, and GC content the clean data were calculated. All the downstream analyses were based on the clean data with high quality.

### Single-Molecule Real-Time Reads Processing

Sequence data were processed using the SMRTlink 7.0 software (PacBio). Circular consensus sequences (CCSs) were generated from the subread BAM files. The CCS.BAM files were used as the output, which were then classified into full-length and non-full-length reads using Lima (PacBio), and polyA sequences were removed using refine (PacBio). The full-length fasta files produced were then subjected to isoform-level clustering [n*log(n)]. Additional nucleotide errors in consensus reads were corrected using the Illumina RNAseq data using the software LoRDEC (V0.7;-k 23;-s3) (Salmela and Rivals, 2014). Any redundancy in the corrected consensus reads was removed using CD-HIT (v4.6.8; -c0.95; -T6; -G0; -aL0.00; -aS0.99; -AS30) (Fu et al., 2012) to obtain final transcripts for subsequent analysis.

### Functional Annotation of Genes

Non-redundant transcript sequence obtained after CD-HIT deduplication were regarded as genes and were grouped and mapped to seven protein and nucleic acid databases to obtain gene annotation information. These databases included the NCBI non-redundant protein sequence database (NR) (Li et al., 2002), NCBI nucleotide sequences (Nt), Protein family (Pfam) (Finn et al., 2016), Clusters of Orthologous Groups of proteins and euKaryotic Ortholog Groups (KOG/COG) (Tatusov et al., 2003), a manually annotated and reviewed protein sequence database (Swiss-prot) (Bairoch and Apweiler, 2000), Gene Ontology (GO) (Ashburner et al., 2000) and Kyoto Encyclopedia of Genes and Genomes (KEGG) (Kanehisa et al., 2004). For BLAST searching, we set the e-value to “1e-5” in the NT database analysis and used the Diamond BLASTX software (v0.8.36) and set the e-value to “1e-5” for the NR, KOG, Swiss-Prot, and KEGG analyses.

### Long Non-coding RNA (lncRNA) Prediction

PLEK (version 1.2)^2^ (Li et al., 2014) and CNCI (version 2)^3^ (Sun et al., 2013) were used to predict the gene coding potential of transcripts obtained after CD-HIT deduplication. Then, genes predicted from PLEK and CNCI were blast searched against databases of known proteins using CPC (2.0)^4^ (Kang et al., 2017). The transcripts predicted by PLEK, CNCI, and CPC were subjected to hmmscan homologous searching (PfamScan, 1.6)^5^ in the Pfam database. Transcripts that predicted by PLEK, CNCI, and CPC software, and homologous searched with Pfam database were putatively identified as lncRNAs.

### Identification of Differentially Expressed Genes and Functional Categorization

The clean reads from Illumina RNA-Seq were mapped back onto the final transcript sequences from SMRT-Seq by using Bowtie (version V2.3.4, -q;–phred33;–sensitive;–dpad 0;–gbar 99999999;–mp 1,1;–np 1;–score-min L,0,-0.1;-I 1;-X 1000;–no-mixed;–no-discordant;-p 8;-k 30) (Supplementary Table 1). Gene expression levels were calculated by RSEM (V1.3.0, –phred33;-quals;–forward-prob 0.5;–time) with FPKM (expected number of Fragments Per Kilobase of transcript sequence per Millions base pairs sequenced) method. Differential expression analysis of the Ocy_N_L group and the Ocy_A group was carried out using the DESeq2 R package (1.16.1) (Love et al., 2014). DESeq2 provides statistical routines to determine differential expression in digital gene expression data using a model based on the negative binominal distribution.

The resulting *P*-values were adjusted using the Benjamini and Hochberg’s approach to control the false discovery rate. Genes with an adjusted *P*-value < 0.05 found by DESeq2 were assigned as differentially expressed.

### Gene Ontology and Kyoto Encyclopedia of Genes and Genomes Analysis of Differentially Expressed Genes

Gene Ontology (GO) enrichment analysis of Differentially Expressed Genes (DEGs) was implemented by the GOseq (version 1.10.1),^6^ in which gene length bias was corrected. GO terms with corrected *P*-value less than 0.05 were considered significantly enriched by the DEGs. KEGG is a database resource for understanding high-level function and utilities of the biological systems, such as cells, organisms, and ecosystems, from molecular level information, especially large-scale molecular datasets generated by genome sequencing and other high-throughput experimental technologies.^7^ We got KOBAS software (version 3.0)^8^ to test the statistical enrichment of DEGs in KEGG pathways.

### Protein-Protein Interaction Network Construction and Hub Gene Identification

The PPI network for the *O. cynotis* gene products was predicted using Search Tool for the Retrieval of Interacting Genes (STRING, version 11.0)^9^ online database. The PPI network was analyzed using Cytoscape software (version 3.5.1),^10^ which also calculated the degree of each protein node. The genes with the top 10-degree scores were identified as hub genes.

### Analysis of Allergen Genes

The 5,431 final transcripts of *O. cynotis* demonstrated from SMRT-Seq were BLAST (e-vaule;10e-5) searched against allergen sequences from the allergome database website^11^ to obtain homologous sequence. These homologous sequences can be regarded as putative allergen genes (Chruszcz et al., 2018). The sequence homologs of known mite allergens and the DEGs were filtered from the putative allergen genes.

### Quantitative Real-Time Reverse Transcription PCR Identification

Sixteen genes were selected to validate their expression levels using quantitative real-time reverse transcription PCR (qRT-PCR). Among these genes, five were hub genes from PPI network analysis, and eleven genes are putative allergen genes with high FPKM values in the Ocy_N_L and Ocy_A groups. The qRT-PCR analysis was carried according to previous research (He et al., 2019).

## Results

### Transcriptome Analysis Using PacBio Sequel

The full-length transcriptome of *O. cynotis* mite was generated by PacBio Sequel. In total, 12,842,815 subreads with an average of 1,417 bp from 18.2 Gb of data were obtained. Then 191,632 CCS were generated after removing adapters and artifacts, including 113,064 full-length non-chimeric (FLNC) reads. The length of the FLNCs ranged from 50 to 14,775 nt, with an average of 1,679 nt. The number of polished consensus sequences was 14,210, and the mean length of polished consensus sequences was 1,653 nt (range = 57–8,193 nt). After removing redundant sequences from the corrected consensus reads by CD-HIT, 5,431 final transcripts with an average of 1,774 nt were obtained for subsequent analysis.

### Functional Annotation of Genes

**FIGURE 2:**
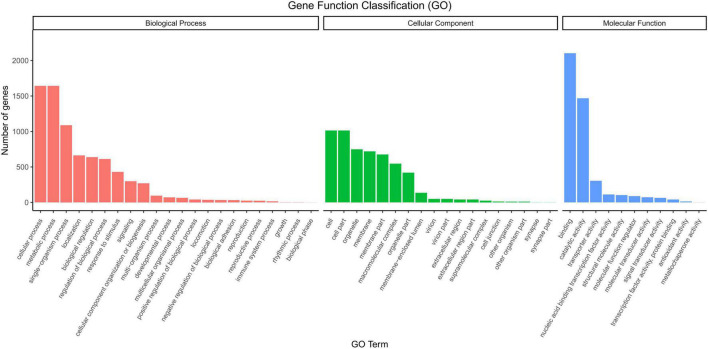
Gene Ontology (GO) functional annotation of *O. cynotis* genes.

A Venn diagram of the gene functional annotation from five databases is given in Supplementary Figure 1. The diagram shows the transcript numbers annotated in the different databases, and the overlapping relationship between these five databases. A total of 1,260 transcripts were annotated in the Nt database; 3,683 in the Pfam database; 3,692 in the KOG/COG database; 3,908 in the Swiss-prot database, and 3,684 and 4,128 in GO and KEGG databases (Supplementary Figure 2), respectively. In the GO annotation, 5,510; 7,723; and 4,369 genes were annotated in the cellular component, biological process, and molecular function categories, respectively (Figure 2). The homologous species of *O. cynotis* were predicted by sequence alignment based on hits from the NR database. Of all the gene hits to NR from BLASTx, the transcripts had the highest number of hits to *Sarcoptes scabiei* (3,138 genes), followed by *Rhagoletis zephyria* (711 genes), and *Tetranychus urticae* (172 genes) (Supplementary Figure 3).

### LncRNA Prediction

We identified 406 lncRNAs of 5,431 genes (S1), as showed in the Venn diagram of the number of LncRNAs predicted according to CNCI, Pfam, Plek, and CPC analyses (Supplementary Figure 4). Two of these lncRNAs were nearly 3,000 nt. The functions of these lncRNAs will be characterized in a future study.

### Gene Expression Level Analysis

**FIGURE 3:**
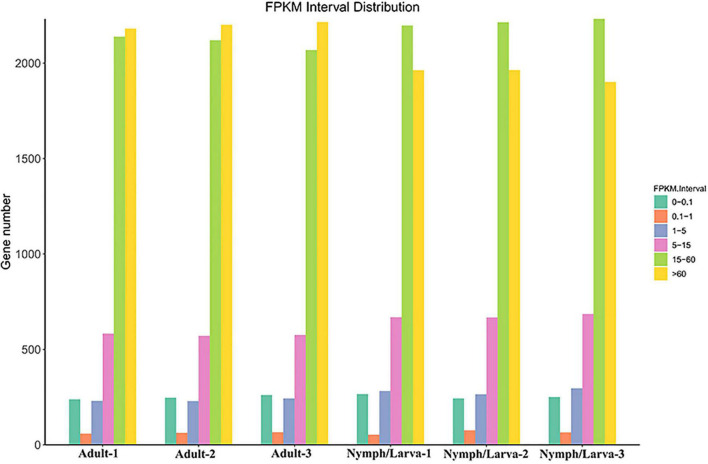
The gene numbers for different Fragments Per Kilo-base of transcript per Million mapped reads (FPKM) values of adult mites *vs*. nymph/larva mites.

To assess the changes in gene expression levels in the different life stages of *O. cynotis*, Illumina sequencing clean reads were compared to the set of final transcripts obtained from PacBio sequencing. Genes expression levels were quantified using Fragments Per Kilo-base of transcript per Million mapped reads (FPKM). The number of genes with different FPKM values from six samples is shown in Figure 3. Over 40% genes in the adult mites had a highest FPKM of more than 60, whereas, the FPKM values of more than 40% of the nymph/larva mites genes were located between 15 and 60. To evaluate the reproducibility of the RNA-Seq data from the three biological replicates for each sample, the Pearson correlation analysis was conducted using the *R*^2^ values of all six samples. The average correlation of gene expression level among biological replicates were 0.9733 and 0.9697 in adult and nymph/larva mites, respectively, which demonstrated good reproducibility of the gene expression results.

### Identification of Differentially Expressed Genes and Functional Categorization

**FIGURE 4:**
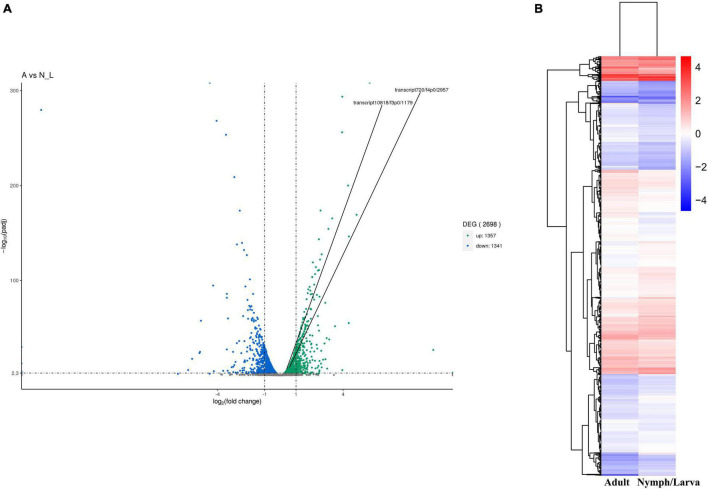
**(A)** Map of the number of differentially expressed genes. **(B)** Cluster map of DEGs.

**FIGURE 5:**
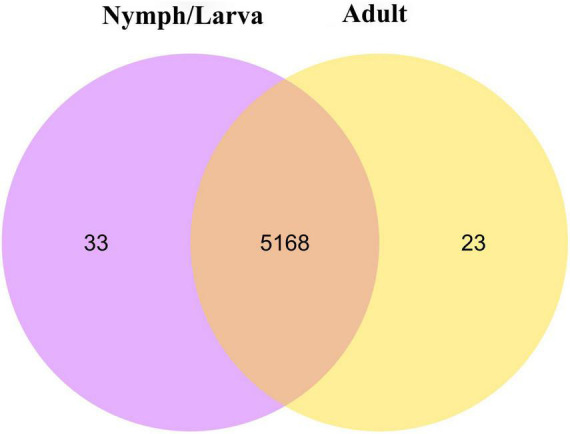
A Venn diagram of differentially expressed genes (the criteria for differential gene screening are |log2(FoldChange)| > 0&qvalue < 0.05).

We identified 2,698 DEGs, among which 1,357 genes were up-regulated and 1,341 genes were down-regulated (Figure 4A). A heat map of all the DEGs was established (Figure 4B). A Venn diagram showed that 5,191 and 5,201 genes were expressed in adult mites and nymphs/larvae mites, respectively. Moreover, 23 and 33 genes were expressed only in adult mites and nymphs/larvae, respectively (Figure 5).

### Gene Ontology and Kyoto Encyclopedia of Genes and Genomes Analysis of Differentially Expressed Genes

**FIGURE 6:**
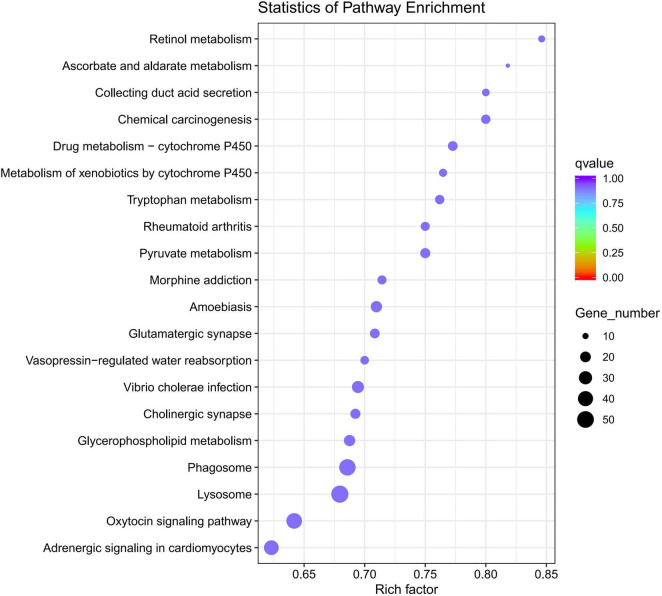
DEGs-enriched Kyoto Encyclopedia of genes and genomes (KEGG) pathways scatter diagram (*q*-value is the *p*-value corrected by multiple hypothesis test, the value range of *q*-value is [0,1]).

Gene Ontology analysis of DEGs indicated that 1,471 DEGs were enriched in the biological process category, 384 DEGs were enriched in the cellular process category, and 785 DEGs were enriched in the molecular process category. Intriguingly, in the molecular process category, more than 500 DEGs were associated with the term “catalytic activity” and over 200 DEGs were associated with the term “hydrolase” activity. In the biological process category, DEGs were mainly associated with the terms “single-organism metabolic process” and “proteolysis.” The main KEGG pathways associated with the DEGs were “Lysosome,” “Phagosome,” “Drug metabolism -cytochrome P450” and “Rheumatoid Arthritis” signaling pathways. DEGs-enriched KEGG pathways scatter diagram is shown in Figure 6.

### Protein-Protein Interaction Network Construction and Hub Gene Identification

The PPIs among final transcripts from PacBio sequencing and DEGs were predicted using STRING tools. The analysis identified 49 nodes and 147 edges among the final transcripts; and 26 nodes and 47 edges among the DEGs. The results showed that FBpp0079187 and FBpp0085265 were both hub genes among final transcripts and DEGs. The hub genes are listed in Supplementary Table 2.

### Analysis of Allergen Genes

According to the results from the putative allergen analysis, 397 putative allergen genes were obtained from the final transcripts, among which 231 were DEGs. Seventy-seven sequences were homologs of known mite allergens from *Dermatophagoides farinae*, *Dermatophagoides pteronyssinus*, *Tyrophagus putrescentiae*, *Euroglyphus maynei*, and *Lepidoglyphus destructor*.

### Quantitative Real-Time Reverse Transcription PCR Validation of Differentially Expressed Genes

**FIGURE 7:**
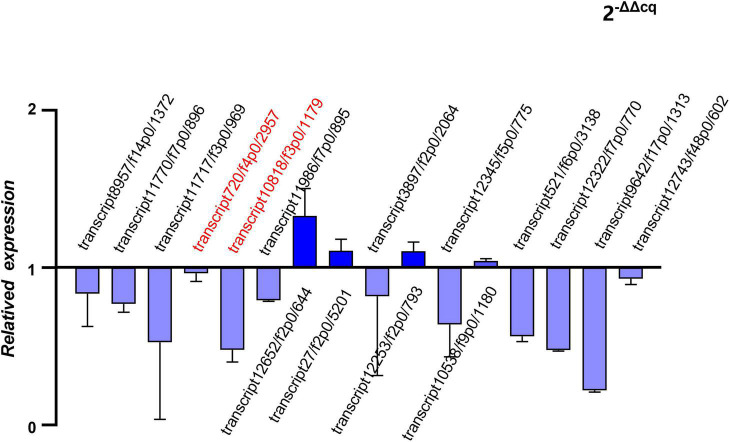
Quantitative real-time reverse transcription PCR (qRT-PCR) of hub genes and putative allergen genes (relative expression of Ocy_N_L groups to Ocy_A groups).

Five hub genes and 11 putative allergen genes were selected to validate their expression levels using qRT-PCR. Furthermore, a comparison between the expression value of qRT-PCR and the FPKM was conducted. Two of these hub genes were all outstanding genes with a high degree of connectivity in the PPI network among the final transcripts and DEGs. Selected putative allergen genes were from those with the top 30 FPKMs in both of adult mites and nymphs/larvae. The relative expression levels of these sixteen genes are presented in Figure 7. The results of the analysis indicated that the expression levels of selected genes determined using RNA-Seq were reliable.

## Discussion

In this study, a combination of SMRT and Illumina sequencing was applied to identify DEGs between the different development stages of *O. cynotis*. Illumina sequencing is limited to the assembly of full-length transcripts, whereas SMRT-Seq overcomes these limitations by mostly generating the full-length transcript as one read (Rhoads and Au, 2015). Bioinformatic tools, such as LoRDEC and CD-HIT could rectify the errors of SMRT reads (Li et al., 2017; Xiao et al., 2017). After SMRT sequencing and processing, the maximum length of final transcripts was 8,193 nt and the N50 of these transcripts were 2,059 nt, with an average of 1,774 nt. Functional annotation analysis showed that the transcripts had the highest number of hits to *S. scabiei* in NR BLASTx, probably because *S. scabiei* and *O. cynotis* are mites that both belong to Arthropoda, Arachida, Arcarina. However, *S. scabiei* belongs to the Sarcoptidae and *O. cynotis* belongs to the Psoroptidae.

LncRNAs are key molecules that regulate gene expression and have become a hot topic in biology (Luo et al., 2017; Jia et al., 2018). To accurately predict lncRNAs, PLEK and CNCI were used to predict the gene coding potential of the final transcript sequences, identifying 4,831 and 4,624 transcript sequences, respectively. Then, 707 and 1,600 transcript sequences were obtained from CPC and Pfam database analyses. In the Pfam database, Pfam-A database records the high-quality domains of most known proteins and the Pfam-B database covers the domain families more comprehensively, which made the gene coding potential prediction more accurate. Ultimately, 406 lncRNA sequences were predicted. Supplementary Figure 5 shows that the average length of the mRNAs was 8,193 nt, whereas that of the lncRNAs was 3,377 nt. The functions of these putative lncRNAs require further study.

DEGs analysis identified 23 genes that were unique to adult mites and 33 genes that were unique to nymphs/larvae. Among the genes unique to adults, functional annotation predicted that three of them encoded autophagy protein 6, colincin immunity protein, and acetylcholinesterase. Among the genes unique to nymphs/larvae, functional annotation predicted that three of them encoded protein kinase domain, cystathionine beta-synthase-like, and major facilitator like protein 5. However, some of these unique genes had no annotation in the seven databases. These genes might have crucial functions in the development, reproduction, and pathogenic mechanism of the different life stages of *O. cynotis* mites.

Differential gene expression analysis identified 2,698 DEGs (1,357 up-regulated and 1,341 down-regulated) between adult mites and nymphs/larvae. Gene Ontology analysis of the DEGs indicated that genes involved in catalytic activity, hydrolase activity and proteolysis might play a significant role in the life stages of mites. Proteases are the main allergens of the house dust mite, and the hydrolysis of these proteases is reported to promote and aggravate the allergic reaction and inflammatory responses of the host (Stewart and Robinson, 2003). In *Psoroptes ovis* (*P. ovis*) infections, proteases can degrade fibrinogen to provide a flow of serous exudate from the host (Kenyon and Knox, 2002). For *S. scabiei* infections, aspartic protease can digest serum molecules and skin from the host (Mahmood et al., 2013). Equally, hydrolases might also help to mite survival and invasion. The enrichment of the DEGs in proteolysis and hydrolase activity could explain how the mites in different life stages have different abilities to invade host skin, i.e., the hydrolases and proteases might participate in the invasion of *O. cynotis* mites.

In the KEGG pathway analysis of DEGs, the up-regulated genes were enriched in 243 pathways and the down-regulated genes were enriched in 231 pathways. The KEGG pathways are illustrated in Figure 6, including both up-regulated and down-regulated genes. These pathways were “Lysosome,” “Phagosome,” and “Drug metabolism Cytochrome P450” signaling pathways; however, the top pathway enriched for the down-regulated genes was the oxytocin signaling pathway (30 DEGs). The hub genes selected in the PPI network might have pivotal roles in the development of *O. cynotis* mites. FBpp0079187 and FBpp0085265 were hub genes defined in both the final transcripts and DEGs. FBpp0079187 was annotated as receptor of activated protein C kinase 1-like (RACK1-like) in the NR database. RACK1 is a candidate interacting protein for establishing infection of *Bombyx mori cypovirus* (Zhang et al., 2017). RACK1 participates in physiological process, such as development, system function, and circadian rhythm. Moreover, it plays a significant role in shuttling proteins around the cell, anchoring proteins at particular locations, and stabilizing protein activity (McCahill et al., 2002; Adams et al., 2011). FBpp0085265 was annotated as elongation factor 2-like (eEF2-like) in the NR database. eEF2 has an essential role in protein synthesis, in which it catalyzes the translocation of tRNA and mRNA (Carlberg et al., 1990; Kaul et al., 2011). These two hub genes were the top two DEGs among the hub genes, which suggested that the main difference between adult mites and nymph/larva mites was in the life processes of development system function and circadian rhythm.

*O. cynotis* mites are the most common etiological agent of otitis externa in cats and dogs, which feed on lymph, tissue fluid, and blood of the host. When mites invade a host, dermatitis or allergic reactions occur. Allergic reactions can be protective, but can also be harmful to the host, leading to tissue damage and immunological hypersensitivity. The ability of an antigen to induce allergic sensitization is called allergenicity (Thepen et al., 1992; Hales et al., 2006; Chapman et al., 2007; Tsolakis et al., 2018). Allergens of mites could promote inflammation and allergic reactions, including rhinitis, asthma, and dermatitis (Aalberse, 2000; Arlian, 2002; Stewart and Robinson, 2003). In the present study, 231 transcript sequences were predicted to be homologous genes of allergens, and 77 of them were homologous genes of known mite allergens. These genes might be the main participants in the pathogenesis of *O. cynotis* mites. The role played by allergens in house dust mite infections has made them a hot topic in mite research. House dust mite allergens were identified to increase the allergic immune response of the host by cleaving IgE receptors and boosting the innate immune response (Gough et al., 1902; Trompette et al., 2008; Mizuma et al., 2016). Twenty-one house dust mite allergens have been widely studied, including their structure, chemical and biological properties (Thomas et al., 2010; Fischer and Walton, 2014). Several other allergens were predicted in the genome or transcriptome of *S. scabiei* and *P. ovis*, such as triosephosphate isomerase, chitinase-like protein, *Pso* 1, *Pso* 2, *Pso* 10, and *Pso* 11 (Temeyer et al., 2002; Nisbet et al., 2006, 2007; He et al., 2017, 2018; Korhonen et al., 2020). Moreover, a series of sequences homologous to known mite allergens were predicted in the transcriptome of *Chorioptes texanus* (He et al., 2019). These findings highlight that mite allergens might also play a crucial role in the pathogenesis of chorioptic mange. In the present study, the allergens selected for qRT-PCR verification were homologous to mite allergens, such as Der p 2, Ferritin, Fatty acid-binding protein, Tropomyosin, High molecular weight allergen M-177, Der f 30 allergen, Group 14 allergen protein, HDM allergen, Der f 13 allergen, Eur m 1, and Der f 39. In total, we predicted 77 genes in *O. cynotis* mites that were homologous to known allergen sequences; however, the functions of these sequences require a further investigation. Research concerning allergens from the house dust mite, *S. scabiei*, *P. ovis* and other mites will provide the basis for further research into *O. cynotis* allergens.

## Data Availability Statement

The data presented in the study are deposited in the NCBI repository, accession number PRJNA713931.

## Ethics Statement

This study was approved by the Animal Care and Use Committee of Sichuan Agricultural University (SYXK2019-187).

## Author Contributions

GY and RH conceived and designed the experiments. RH and QZ performed the experiments. RH and GY analyzed the data. RH wrote the manuscript. GY, RH, QZ, XG, YX, JX, and XP prepared the samples. All authors contributed to the article and approved the submitted version.

## Conflict of Interest

The authors declare that the research was conducted in the absence of any commercial or financial relationships that could be construed as a potential conflict of interest.

## Publisher’s Note

All claims expressed in this article are solely those of the authors and do not necessarily represent those of their affiliated organizations, or those of the publisher, the editors and the reviewers. Any product that may be evaluated in this article, or claim that may be made by its manufacturer, is not guaranteed or endorsed by the publisher.

## Footnotes

1
http://www.nc3rs.org.uk/arrive-guidelines

2
https://sourceforge.net/projects/plek/

3
https://github.com/www-bioinfo-org/CNCI

4
http://cpc2.gao-lab.org/download.php

5
https://www.ebi.ac.uk/seqdb/confluence/display/THD/PfamScan

6
http://www.bioconductor.org/packages/release/bioc/html/goseq.html

7
http://www.genome.jp/kegg/

8
http://bioinfo.org/kobas/download/

9
http://string-db.org/

10
https://cytoscape.org

11
http://www.allergome.org/

## Supplementary Material

Supplementary Figure 1 A Venn of gene functional annotation.

Supplementary Figure 2 Gene function annotation using Kyoto Encyclopedia of genes and genomes (KEGG) pathway classification.

Supplementary Figure 3 Homologous species distribution of *O. cynotis* annotated in the non-redundant (NR) database.

Supplementary Figure 4 Venn diagram of the number of long non-coding RNAs (LncRNAs) predicted by CNCI, Pfam, Plek, and CPC.

Supplementary Figure 5 Long non-coding RNA (LncRNA) and mRNA length distribution map.
