# Supplementary figures and images for: Transcriptome Analysis of Otodectes cynotis in Different Developmental Stages
Source: Front Microbiol. 2022 Apr 4;13:687387. doi: 10.3389/fmicb.2022.687387 (PMC9014205; doi:10.3389/fmicb.2022.687387)

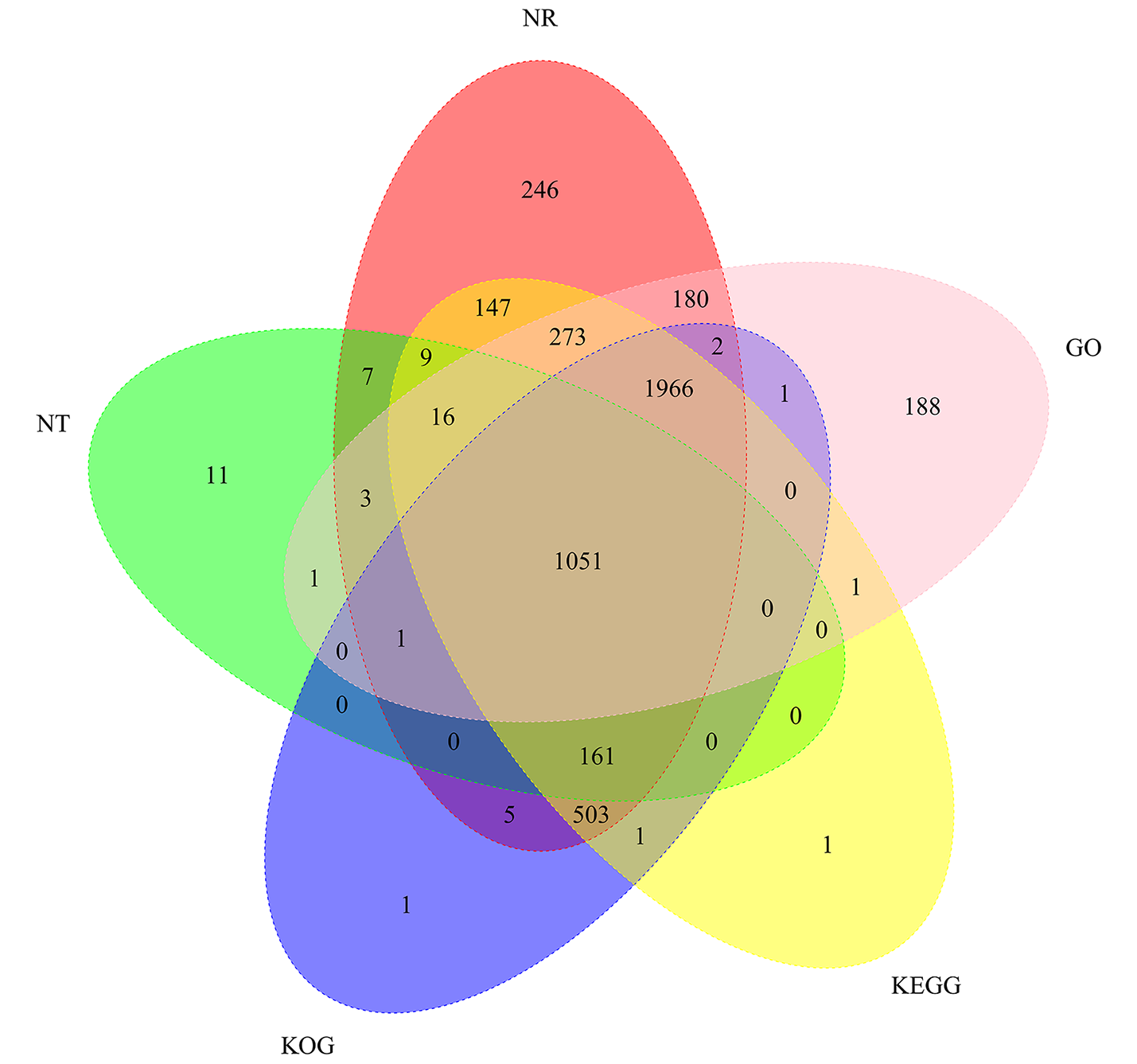

Supplement: Supplementary Figure 1 — A Venn of gene functional annotation. [file Image_1.TIF]

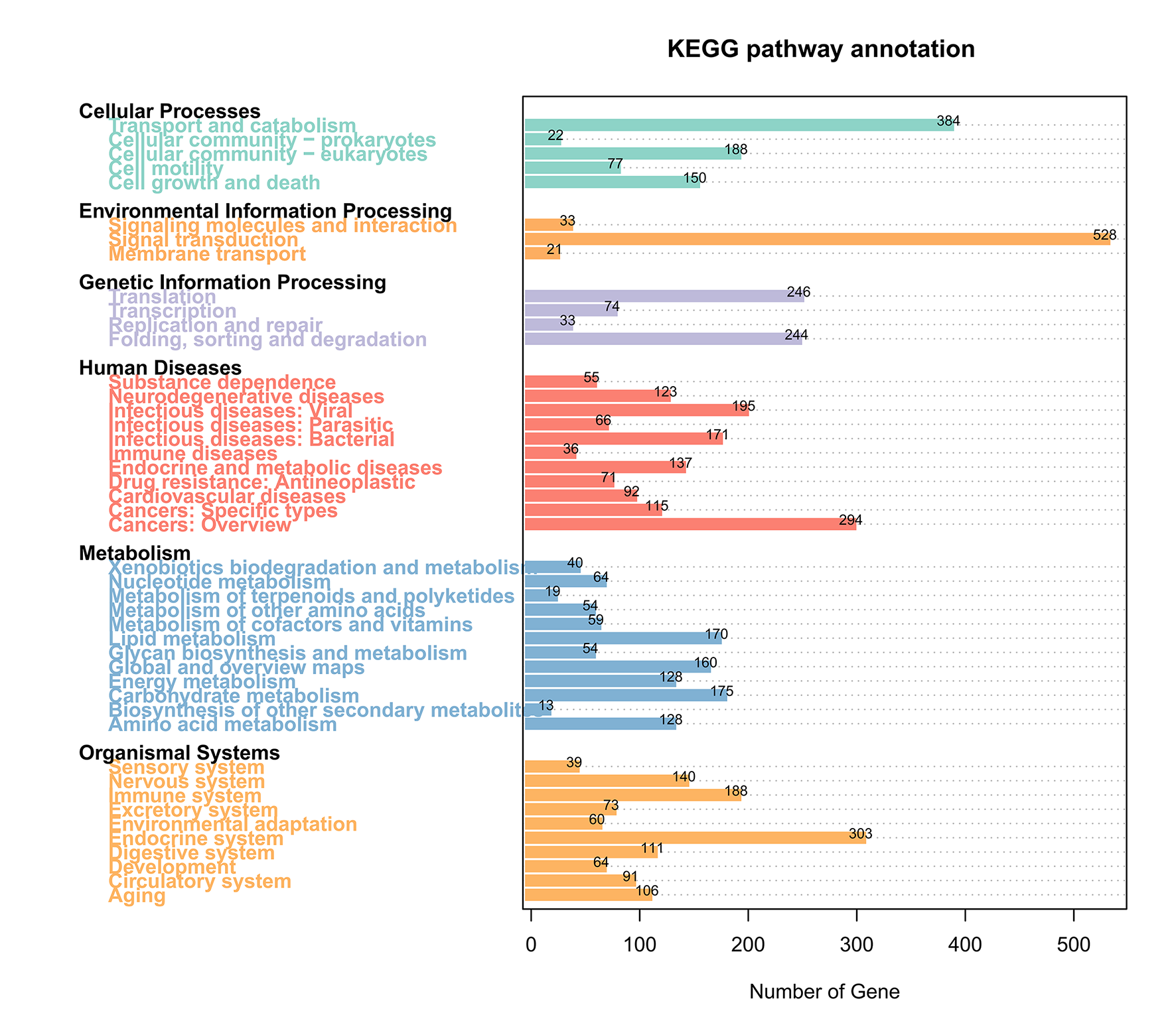

Supplement: Supplementary Figure 2 — Gene function annotation using Kyoto Encyclopedia of genes and genomes (KEGG) pathway classification. [file Image_2.TIF]

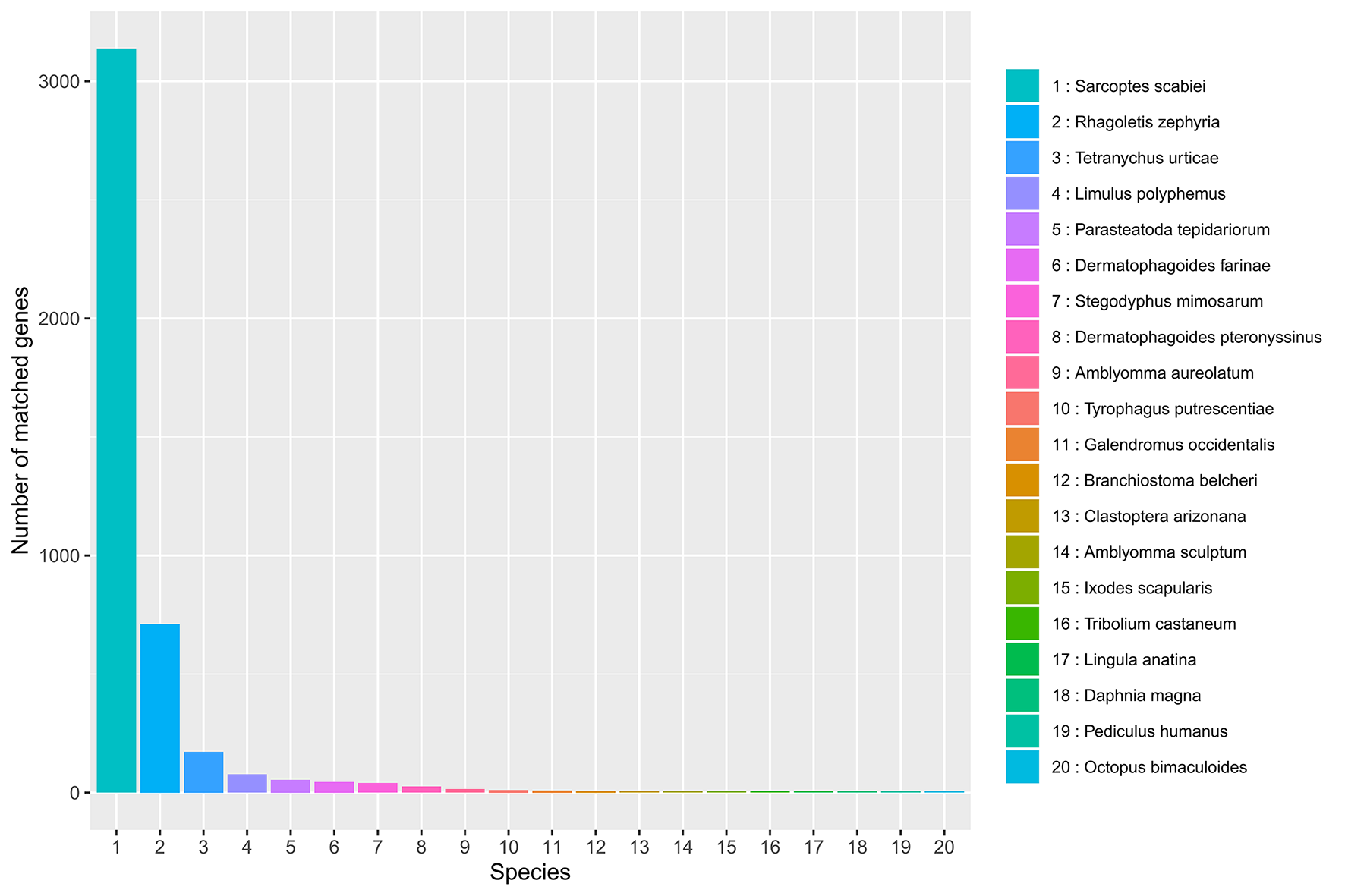

Supplement: Supplementary Figure 3 — Homologous species distribution of O. cynotis annotated in the non-redundant (NR) database. [file Image_3.TIF]

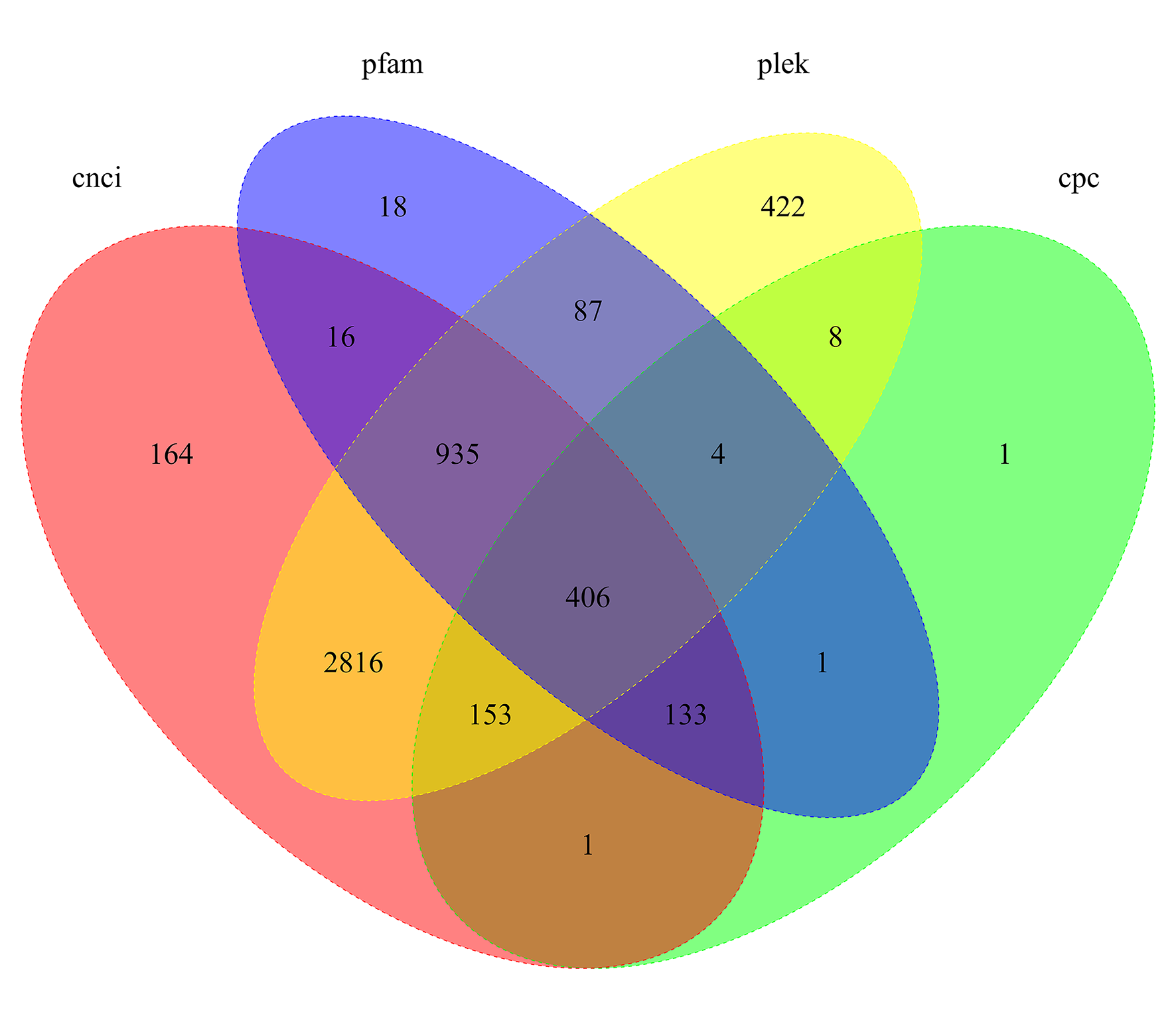

Supplement: Supplementary Figure 4 — Venn diagram of the number of long non-coding RNAs (LncRNAs) predicted by CNCI, Pfam, Plek, and CPC. [file Image_4.TIF]

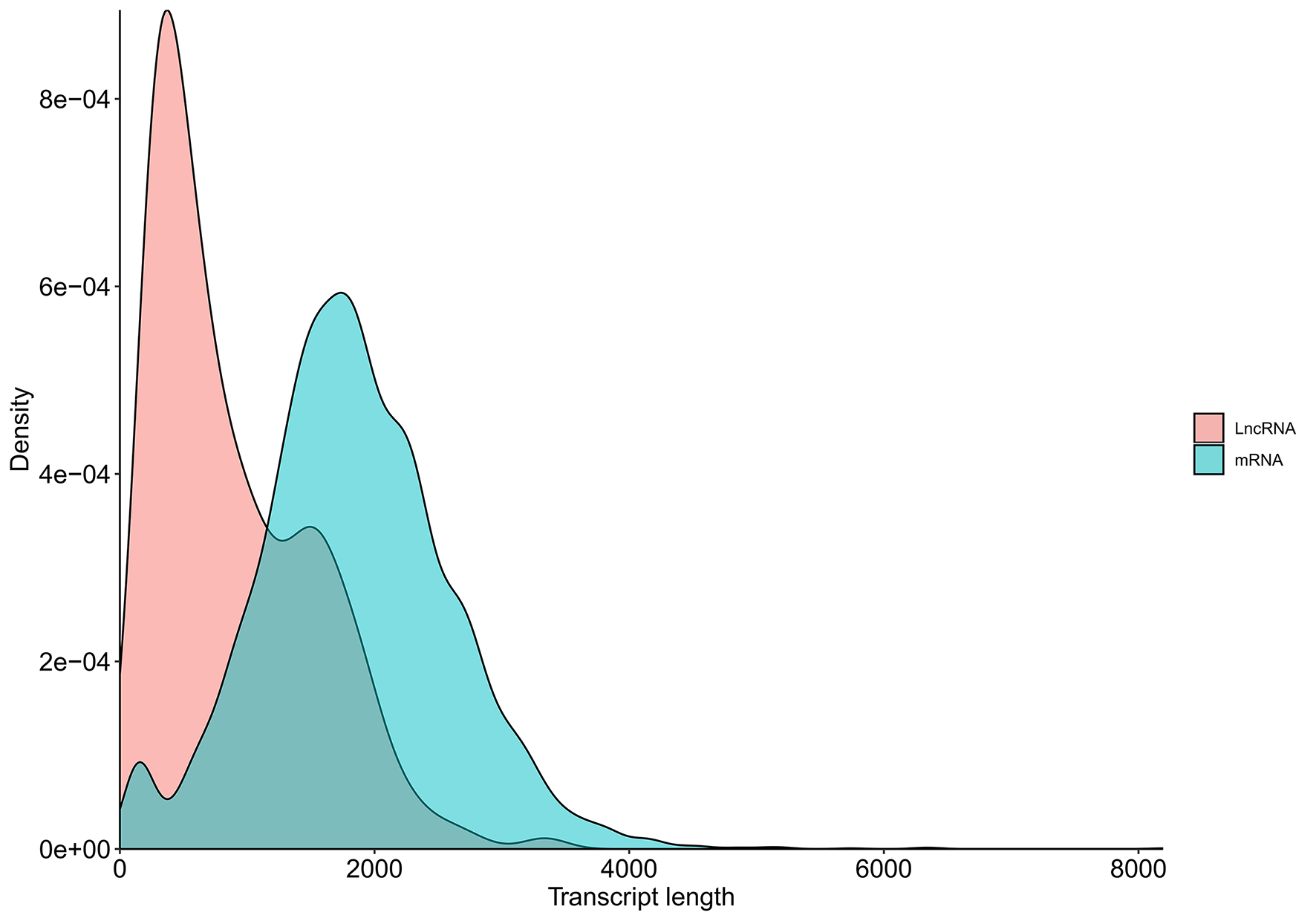

Supplement: Supplementary Figure 5 — Long non-coding RNA (LncRNA) and mRNA length distribution map. [file Image_5.TIF]
